# Supplementary material for: Are carbon emissions trading and green financial instruments synergistic? -Comprehensive quantitative research based on content analysis
Source: PLoS One. 2024 Mar 7;19(3):e0298601. doi: 10.1371/journal.pone.0298601 (PMC10919601; doi:10.1371/journal.pone.0298601)
Supplement: S1 Data — (ZIP) [file pone.0298601.s001.zip › Data/地级市Z_F工作报告.zip!/─■╧─/╥°┤¿╩╨/└┤╫╘╣1⁄2╓┌║┼í╛┬φ┐╦╩2╛▌═°í┐_2019.docx]

各位代表：

现在，我代表市人民政府向大会作政府工作报告，请予审议，并请市政协委员和其他列席会议的同志提出意见。

一、2018年工作回顾

刚刚过去的一年，是全面贯彻党的十九大精神的开局之年，也是银川推动高质量发展极不平凡的一年。一年来，在改革开放40周年、自治区成立60周年历史鼓点下，在习近平总书记“建设美丽新宁夏、共圆伟大中国梦”大庆题词感召下，在自治区党委、政府和市委的坚强领导下，市政府深入学习贯彻习近平新时代中国特色社会主义思想和党的十九大、自治区第十二次党代会精神，团结带领全市人民，狠抓“三转一高”工作落实，令出惟行，锐意进取，攻坚克难，推动银川发展在新征程上呈现新气象。自治区成立60周年大庆各项任务圆满完成，“绿色、高端、和谐、宜居”城市发展新理念全方位实施，银川都市圈、“四区四园”建设等重大战略布局全面铺开，城乡西线供水等一批战略性经济民生工程谋定而动，防范化解重大风险、精准脱贫、污染防治“三大攻坚战”扎实推进，中央巡视、国务院大督查和环保督查反馈问题加快整改，荣获全球首批国际湿地城市称号，获评2018中国最具生态竞争力城市、“国家公交都市建设示范城市”，被列为全国“互联网+教育”和“互联网+医疗健康”示范区核心区，成功举办“一带一路”国际葡萄酒大赛、全球智慧城市峰会，银川知名度、美誉度、影响力持续攀升。地区生产总值同比增长7.2%,规模以上工业增加值同比增长7.5%；固定资产投资同比下降21.9%，社会消费品零售总额同比增长4.8%；一般公共预算收入同口径增长8.0%；城乡居民人均可支配收入分别同比增长7.9%和8.2%。

一年来，重点抓了以下几方面工作：

（一）加快动能转换，高质量发展迈出新步伐。坚持以供给侧结构性改革为主线，出台促进投资10条、工业转型升级24条、“1+2+7”人才新政等一揽子政策，经济结构优化升级。大力实施创新驱动战略，组建葡萄酒产业技术研究院等4个“产学研建”平台，国家高新技术企业达到95家，全社会R&D投入强度达到1.71%。扎实推进“三去一降一补”，淘汰落后产能78万吨，万元GDP能耗下降5%以上，商品房去库存周期缩短至10.78个月，累计减免各项税费130.34亿元。大力推进园区优化整合，经开区跻身国家级开发区百强，中关村双创园获评自治区级现代服务业集聚区，丝路经济园、苏银产业园、军民融合产业园建设初见成效，园区成为引领高质量发展主战场。培育“专精特新”中小企业60家、示范企业13家、自治区标杆企业10家，石墨烯新材料项目一期投产，预计装备制造业增加值增长16%；葡萄酒产业链由种植、加工向创意研发、品牌塑造拓展；电子商务网络零售交易额增长20%。旅游总收入突破150亿元，同比增长15.83%。服务业对经济增长贡献率达到60%。招商引资到位资金505亿元，民营企业占87.5%。投资结构进一步优化，民间投资比重上升至57.7%。

（二）重拳铁腕治污，生态环境建设取得新突破。深入实施“生态立市”战略，以前所未有的决心和力度，投入资金61亿元，加强生态环境治理，生态环境质量持续好转。中央环保督察交办2017年整改任务全部完成，2018年整改任务基本完成，中央环保督察及“回头看”转办件办结率分别达99%、98%，严肃查处了泰瑞制药违规违法生产等问题，困扰群众多年的“异味扰民”问题得到有效解决。“东热西送”一期工程建成投用，集中供热覆盖区域燃煤锅炉应拆尽拆，结束了长期以来低效分散的供热历史。治煤、治车、治企、治尘、秸秆禁烧工作持续发力，空气优良天数较去年增加21天，PM10下降17.9%，PM2.5下降20.8%，空气质量改善幅度位列全国169个重点城市第4、全区第1。工业园区污水实现集中处理，城市污水处理厂全部达到一级A排放标准，清污分流项目建成投运，城市黑臭水体基本消除，城市集中饮用水水源地水质100%达到或优于Ⅲ类标准。6条入黄排水沟人工湿地和滨河湿地扩整连通工程初步建成，新增水域1万亩，黄河银川段水质实现“Ⅱ类进Ⅱ类出”。国家自然保护区生产企业全面清除，灵武再生资源循环园区整治基本完成。全市累计生态修复面积47万平方米，植树造林7.7万亩。

（三）加强统筹协调，银川都市圈建设取得新进展。深入实施区域协调发展战略和乡村振兴战略，“三市一地”携手共进、融合发展。参与实施银川都市圈项目75个，银西高铁、包银高铁、火车站客运综合枢纽等互联互通项目加速推进，高端装备制造、现代纺织等产业协作更加密切，贺兰山东麓绿化、葡萄酒长廊生态防护等生态共治项目进展顺利，教育医疗等领域惠民合作拓展延伸。城市设计等国家改革试点不断深化，凤凰北街打通、贺兰山西路拓宽等16条市政道路建成投用，“疏堵提畅”工程成效明显。建成小微公园10个，人均公园绿地面积达到16.79平方米。生活垃圾分类处理覆盖60万人口，建成投用综合执法平台等智慧城市项目9个，城市品质持续提升。新建水稻、蔬菜“五优”示范基地20个，设施园艺达32万亩，60%以上蔬菜外销，乡村旅游实现营业收入4.8亿元。加快农村人居环境整治，排查整治“大棚房”6853个，清理违法违规占用土地449.3亩。累计建成美丽村庄104个、全国特色小镇3个。

（四）扩大开放合作，改革创新取得新成果。以庆祝改革开放40周年为契机，在更大范围更宽领域扩大开放。深化东西合作，闽宁协作成果入选全国“决胜2020脱贫攻坚展”，献礼影片《闽宁镇》在全国上映；深化政企合作，与中铁、中冶、中电建等多家央企建立战略合作关系；京银、苏银、军地合作不断深化。创新审批模式，“不见面”办理事项达82%，企业开办一日办结，国务院大督查通报银川开办企业指标位列全国第2位、获得信贷位列第7位、工程建设项目报建位列第8位，新登记市场主体4.22万家、增长12.4%。城市管理综合执法体制改革试点全面完成。组建银川高新技术产业投资母基金、民营企业扶持基金，助贷规模达到60.17亿元。申请置换地方政府存量债务52.51亿元，解除政府购买服务协议30个40.6亿元，偿还债务30.73亿元。国际空港三期建成投用，河东国际机场年旅客吞吐量突破900万人次。国际货运班列常态化运营，公铁物流港被确定为国家多式联运示范工程。银川被列为23个空港型国家物流枢纽承载城市之一、55个商贸服务型国家物流枢纽承载城市之一。新设外资企业24家。

（五）加大民生投入，人民群众福祉有了新收获。财政支出80%以上用于改善民生。争取各级财政扶贫资金5.2亿元，实施产业扶贫项目58个，全面完成“十三五”易地扶贫搬迁安置任务，16个贫困村6732名贫困人口脱贫退出。创业带动就业1.97万人，农村劳动力转移就业10.58万人，城镇新增就业5.5万人，城镇登记失业率控制在3.58%以内。率先在西部地区将因病、残疾照顾返贫致贫家庭纳入低保，城乡低保标准位居西北省会城市前列。贫困群众大病医保报销比例提高5%。8.15万被征地农民纳入城镇职工养老保险。5182户困难群众住上“暖心房”。“首都带首府”教育医疗合作深入推进。实施北师大银川学校、北塔中学等28个教育项目，新增学位1.44万个。上线全国首个互联网医院监管平台，28家互联网医院累计服务患者900多万人次，“互联网+医疗健康”得到国务院大督查通报表扬。新建基层综合文化服务中心60个、示范性农民文化大院80个，推出《不到长城非好汉》《岩石上的太阳》等一批优秀剧目。2018年中国·意大利版画作品联展、毗邻地区文艺展演、舞剧《月上贺兰》等精品文化“外输”步伐加快，城市文化品牌不断增强。“丝绸之路”国际马拉松赛掀起全民健身新热潮。创建自治区级民族团结进步示范单位20个，列为全国少数民族流动人口服务治理体系建设试点城市。安全生产形势持续好转，防震减灾能力不断提升，成功抗击“5·10”“7·22”等洪灾。打掉涉黑恶势力犯罪团伙20个，命案发案率下降55%，人民群众安全感进一步增强。史志、档案、双拥、气象、妇女儿童、老龄、残疾人、红十字等工作有了新进步。

（六）强化“四个意识”，政府治理能力得到新提升。始终把学习贯彻习近平新时代中国特色社会主义思想和党的十九大精神作为首要政治任务，加强意识形态、脱贫攻坚、生态文明等内容学习，切实增强“四个意识”，坚定“四个自信”，坚决做到“两个维护”。始终在思想上、政治上、行动上与党中央保持高度一致，严格落实党内政治生活制度，严格执行重大事项请示报告制度，坚决执行党中央和自治区党委、政府以及市委的重大决策部署，始终做到上级有决策、政府见行动。严格落实“一岗双责”，健全完善政府党组议事规则等制度，深入推进违反中央“八项规定”精神突出问题专项治理，开展“电视问政”12期，问责“三不为”干部191人，政府系统党风廉政建设全面深化。加强法治政府建设，依法制定行政规范性文件15件，主动公开政务信息14万余条，政府透明度指数位列全国第7位。银川审改经验编入《中国地方法治发展报告》蓝皮书。自觉接受人大和政协监督，办理人大代表议案6件、建议121件，办理政协提案199件，办复率均达到100%。中央第八巡视组移交信访件办结率95%以上，“12345”热线畅通民意诉求通道，市民满意度不断提升。

各位代表，过去的一年，我们负重拼搏、砥砺前行，顶住了一个又一个压力，攻克了一个又一个难关，推动经济社会持续健康发展。一年的成果来之不易，这是习近平新时代中国特色社会主义思想正确指引的结果，是自治区党委、政府和市委坚强领导的结果，是市人大、市政协、社会各界有效监督和大力支持的结果，是全市人民勠力同心、合力攻坚、撸起袖子加油干的结果。在此，我代表市人民政府，向为银川经济社会发展付出辛勤劳动、给予充分理解的全市人民，向给予政府工作大力支持的人大代表、政协委员、各民主党派、工商联、无党派人士和各人民团体，向中央、自治区驻银单位、驻银部队、武警官兵、政法干警，以及所有参与银川建设的劳动者、关心支持银川发展的朋友们，表示崇高的敬意和衷心的感谢！

在看到成绩的同时，我们也清醒地认识到，高质量发展中还面临诸多问题和挑战：一是绿色发展基础还不稳固，污染治理和生态保护任务依然艰巨，绿色生产和生活方式还需加快构建。二是高端发展创新动力不足，产业结构不优、集群度低，企业主体实力不强，有效投资接续不足，新产业新动能培育发展缓慢，政府债务化解压力较大。三是和谐宜居发展结构性问题明显，民生保障水平还有待提高，教育、医疗、养老等公共服务供给总量不足、质量不优，城乡建设、区域发展还不平衡，城市建管水平需要进一步提升，脱贫富民还需持续用力。四是个别干部思想观念、能力作风与高质量发展要求不相适应，营商环境还需优化，行政效率还有较大提升空间。对此，我们一定要高度重视，坚持问题导向，聚焦短板弱项，采取强力措施，切实加以解决。

二、2019年工作

今年是新中国成立70周年，也是全面建成小康社会关键之年，做好今年工作意义重大。当前，国内外形势发生深刻复杂变化，世界面临百年未有之大变局，但我国发展仍处于并将长期处于重要战略机遇期。随着新时代改革开放再出发，“一带一路”建设深入推进，新一轮西部大开发战略深入实施，银川都市圈、沿黄生态经济带等重大决策部署纵深推进，减税降费及加快民营经济发展等利好政策效应充分释放，特别是“高铁时代”即将到来，银川高质量发展当下有为、未来可期。我们必须牢牢坚持发展第一要务，以强烈的担当精神和使命意识，主动对标习近平总书记视察宁夏重要讲话精神和“建设美丽新宁夏、共圆伟大中国梦”的重要指示，主动对标“走在前、作表率”的要求，主动对标“绿色高端和谐宜居”美丽新银川的决策部署，自我加压、勇于奋进、全力攻坚，奋力推进银川高质量发展。

今年政府工作的总体要求是：以习近平新时代中国特色社会主义思想为指导，深入贯彻党的十九大和十九届二中、三中全会及中央经济工作会议精神，全面落实自治区第十二次党代会、十二届六次全会和市委十四届六次、七次全会决策部署，统筹推进“五位一体”总体布局，协调推进“四个全面”战略布局，坚持稳中求进工作总基调，坚持新发展理念，坚持以供给侧结构性改革为主线，继续打好三大攻坚战，持续实施创新驱动、生态立市、脱贫富民、乡村振兴战略，扎实推进经济、政治、文化、社会、生态文明建设高质量发展，以首府责任、首府标准和首府担当，加快建设“绿色高端和谐宜居”美丽新银川，为实现“两个率先”目标打下决定性基础，为建设美丽新宁夏、共圆伟大中国梦贡献力量，以优异成绩迎接新中国成立70周年。

主要预期目标是：地区生产总值同比增长7%左右，总量突破2000亿元大关；规模以上工业增加值同比增长6.5%左右；固定资产投资同比增长3%以上；社会消费品零售总额同比增长6%左右；一般公共预算收入同口径增长7%左右；城镇和农村居民人均可支配收入分别同比增长7%、7.5%。全面完成自治区下达的各项约束性指标。

实现以上目标，困难不容低估。要坚定必胜信念、增强忧患意识、保持战略定力，聚焦建设“绿色高端和谐宜居”美丽新银川，坚持调子不变、频道不换，劲头不松、力道不减，努力做好以下工作：

（一）坚定不移推动绿色发展，加快建设西北地区生态文明先行市。加强生态文明建设、推动绿色发展，是实现高质量发展的题中之义。要深入实施生态立市战略三年行动计划，统筹兼顾、综合施策，推动生态环境质量持续改善，满足好人民群众对优美生态环境的需要。

打好污染防治攻坚战。聚焦环境突出问题，坚守阵地、巩固成果，不折不扣抓好中央环保督察反馈问题整改落实，推动污染防治由被动保护向主动建设转变，从重点突破向全形态、全链条、全市域治理迈进。打好蓝天保卫战。启动实施“东热西送”二期工程，淘汰两县一市集中供热覆盖区域40蒸吨以下燃煤供热锅炉，帮助和督促泰瑞、启元等生物发酵企业整治（搬迁），清理整治“散乱污”企业，抓好秸秆禁烧及综合利用，建成区裸露堆场地面整治全覆盖，城市垃圾、渣土运输全密闭，开展柴油货车污染专项治理，加快淘汰老旧车辆，确保优良天数比例达到78%以上。打好碧水保卫战。层层压实河（湖）长责任，推动“河（湖）长制”向“河（湖）常治”转变。实施滨河水系连通、中小河流治理、入黄排水沟人工湿地等项目，确保入黄和黄河断面水质稳定达标。年内完成饮用水水源保护区内污染源关闭搬迁，加快建设第一再生水厂，扩容提标第四污水处理厂，全面消除城市黑臭水体。打好净土保卫战。有效管控土壤环境风险，加强工业固体废物堆存排查整治和综合利用。实施农业“三减”行动，绕城高速以内所有农田全面禁止使用化肥、农药、除草剂，其他地区使用量降低15%。

加强生态环境保护。严守城镇开发边界、永久基本农田、生态保护三条红线，统筹推进山水林田湖草系统治理，争创国家森林城市。持续实施大规模国土绿化行动，造林13万亩。东部打好新时代黄河保卫战，加快河滩地生产经营活动退出，守护好白芨滩自然保护区生态环境，构建“黄金河岸”生态廊道；中部以东郊生态公园、南郊植物公园、北郊湿地公园、西郊森林公园和动物园为重点，构建生态绿网体系；西部以110国道为轴线，加快贺兰山东麓整体绿化，建设葡萄长廊防护林工程，绿化美化国道两侧和沿线产业园区。力促清水湖、七子连湖、梧桐湖争创国家级湿地公园，湿地保护率达到85%以上。加强自然生态空间用途管制，严格落实领导干部生态环境损害责任追究制，完善生态补偿机制，筑牢生态文明建设制度保障体系。

推动绿色低碳发展。广泛开展节约型机关、绿色家庭、绿色学校、绿色社区和绿色出行等创建行动，启动实施一体化绿色交通建设示范项目，加强充电站、充电桩等基础设施建设，投放500辆新能源公交车，加快城市慢行系统建设，使简约适度、绿色低碳的生活方式成为广大市民的行动自觉。加强绿色供给，稳步推进煤改气、煤改电，持续实施园区低成本化改造，打造一批绿色园区、绿色工厂、绿色产品，加快处置“僵尸企业”，年内淘汰落后产能5000吨以上，全面完成能耗“双控”任务。当好沿黄生态经济带建设排头兵，因地制宜发展“生态+”新业态，支持葡萄种植+文化旅游、石墨烯+水生态治理应用、荒漠治理+休闲运动等生态经济模式，壮大节能环保、清洁生产、清洁能源等产业，通过“绿色+”让产业变“绿”，让“绿”变成产业，让“绿色引擎”提升城市“颜值”、增强发展动能。

（二）坚定不移推动高端发展，加快建设现代化城市。高端发展的本质是高质量发展，重点是牵住产业结构转型升级这个“牛鼻子”。要以深化供给侧结构性改革为主线，深入实施创新驱动战略三年行动计划，在“巩固、增强、提升、畅通”上下功夫，推动经济发展迈向更高层级。

大力推进科技创新。用好“科技支宁”政策，加快沿黄科技创新改革试验区、国家创新型城市建设，强化产业、研发、市场、资本、人才等全要素协同，提高全要素生产率。加强创新平台建设。完善“产学研建”一体化技术创新体系，持续发挥葡萄酒产业技术研究院等创新平台作用，建成运营上海交通大学（银川）材料产业研究院，布局建设大数据、新材料、智能网联汽车等重点实验室、技术创新中心，推动科研机构市场化、企业化运作。强化企业主体地位。落实研发投入后补助等政策，引导企业建立持续稳定科技投入机制，R&D投入强度达到2.12%，实施重大科技创新项目70个以上，培育“专精特新”企业30家以上，力争国家高新技术企业达到110家以上。着力转化科技成果。健全创新成果交易市场体系，建立源头保障、转移供给、收益分配等体制机制，加强中科院产业育成中心建设，打造一批飞地育成平台和离岸孵化器，转移转化重大科技成果不少于10项。办好中国商标节。优化创新创业环境。全面落实国家科技金融试点城市政策，探索知识产权质押融资等投融资新路径。深入推进人才管理改革试验区建设，完善人才分类评价、服务体系，建设人力资源服务产业园，加强急需紧缺人才引进，大力培养各类人才，推动创新跑出“加速度”。

加快产业转型升级。产业强，则经济强。坚持产业园区化、园区项目化、项目企业化，建立“一个产业、一个规划、一批项目、一套政策、一个团队、一支基金”的支撑体系，助推“十大产业”集群化、高端化发展。着力优化产业结构。提升传统产业，加快新技术新业态与羊绒、生物发酵、食品等产业融合，实施30个重大技改项目，促进传统产业“老树发新芽”。提质优势产业，聚焦增链、延链、补链、强链，加快轴承制造小镇、隆基硅7GW单晶电池、汉尧锂离子电池等重点项目建设，做大做强高端装备制造、新能源、新材料等产业。提速新兴产业，启动实施网络强市战略，推动5G网络布局，加快“互联网+”发展步伐，实施比亚迪新能源乘用车等重大项目，推动数字经济、军民融合、新能源汽车等新兴产业尽快壮大，抢占经济发展新高地。提档现代服务业，实施宁安文化园、丝路明珠塔、京东“亚洲一号”电商物流园等项目，发展智慧旅游、“旅游+”、信息消费等新业态，做强“葡萄酒+”复合业态，培育壮大生产性服务业，推动现代物流、文化旅游、健康医养、葡萄酒等产业质量提升、发展提速，厚植经济发展新底色。深化产业园区整合转型。编制完成园区发展规划，明确发展定位，厘清主导产业，年末园区主导产业占比达到50%以上。深化园区赋权改革，确保自治区下放的权限全部到位。深化管理机制改革，苏银产业园、军民融合产业园、中关村双创园引入专业管理团队，建立浮动绩效体系和正负激励机制。贺兰工业园推进“腾笼换鸟”，灵武再生园迁建企业尽早投产达效，永宁工业园加快发酵类企业改造搬迁。“多评合一”“区域评”在园区全面推开，进一步降低企业运营成本。着力扩大精准有效投资。坚持投量投向投效并重，深化重大项目领导牵头包抓、“两个清单”台账销号、“首席服务官”等机制，聚焦人工智能、工业互联网、物联网等重点投向，谋划和储备一批项目，集中力量推动中太镁合金板材、银河大尺寸半导体等重点项目建成达效。更大力度优化投资结构，加大产业投资考核权重，发挥融资担保、助贷基金、产业基金、高新技术产业投资母基金作用，引导各类资本向重点产业、重大项目及新经济、新业态聚集。完善招商引资优惠政策，建立以落地率、到位资金和投入产出比为导向的招商引资评价考核机制，全力开展产业链招商、专业化招商，全面完成自治区下达的任务。加强政策创新。大力实施“引金入银”计划，推进中国进出口银行设立分支机构，组建中小微企业小额贷款公司，积极发展股权投资、天使投资、风投等金融业态，培育多层次资本市场，增强金融服务实体经济能力。注重政策供给的协调性和操作性，完善政策操作方案，建立政策落实督查评价反馈机制，提升政策执行力、知晓率、到位率，推动政策全链条、无缝隙落地见效。

深化改革扩大开放。坚持改革开放“双轮驱动”，着力营造一流营商环境，充分激发发展动力、释放发展活力。培育壮大民营经济。制定支持民营经济发展壮大的政策措施，全面实施市场准入负面清单，鼓励民营企业参与国企改革、进入更多领域。健全企业家参与涉企政策制定机制，实施信用奖惩和黑名单制度，激励和保护企业家精神。严格落实减税降费各项政策，清理规范政府性基金，竭力降低企业负担。树立呵护项目、善待企业风向标，及时为企业纾困解忧，构建“亲清”新型政商关系。深化重点领域改革。全面完成机构改革任务。加快事业单位改革试点。积极稳妥推进综合执法改革。深化“放管服”改革，全面推行审批服务“六办”和“四个一批”，应进未进事项全部进驻大厅集中办理，探索容缺受理审批模式，让企业办事不出园区、居民办事不出社区。完成政务服务标准化试点建设。启动要素市场化配置改革，搭建闲置资源要素共享平台，有效促进资源要素向优质企业、优质项目、优质产业流动。实施改善营商环境三年行动计划，打造公平法治可预期的发展环境。深化国企改革，积极发展混合所有制经济。扩大对外开放合作。主动融入“一带一路”建设，推动银川—德黑兰货运班列常态化运行，优化加密国内国际航线，加强与沿海沿边港口（口岸）对接，深化铁路航空口岸一体化运行、通关一体化合作，推进跨境电子商务综合试验区建设，构建内外畅通、通江达海、多式联运的开放通道。复制自贸区经验，加快进境肉类（水果、种苗）口岸和国际快件监管中心建设，完成公铁物流园海关特殊监管区建设，争取生皮、棉花、黄金委外加工和一般纳税人政策试点，申请恢复开设外汇商品免税店，推广应用国际贸易“单一窗口”，创造更加快捷便利的通关条件。抢抓西部大开发20周年和中阿博览会机遇，强化东西协作、京银合作，深化国际产能合作，对外商投资实行“准入前国民待遇+负面清单”制度，支持企业大力开拓国际市场，加快丝路经济园民营经济聚集高地建设，扎实推进中沙、中阿产业园建设，提升开放型经济发展水平。

防范化解重大风险。坚持“坚定、可控、有序、适度”原则，密切监控、稳妥处置突出风险点，守住不发生系统性、区域性、行业性风险的底线。严控政府债务风险。综合运用股权转让、市场化运作、盘活政府存量资产等方式，妥善处置债务存量。健全政府债务举借审批管理、债务风险和责任追究机制，加强融资平台、政府购买服务等隐性债务监管，坚决遏制隐形债务增量。建立偿债风险准备金，应对突发债务风险。严控金融风险。严控小贷、担保等类金融机构经营风险，高压打击、铁腕治理非法集资、金融诈骗等违法违规金融活动。健全政府产业引导、产业投资等基金投入退出机制，完善担保风险补偿、企业救助等机制，防范企业资金链断裂。严控房地产风险。开展商品房预售资金监管试点，有效防控和处置逾期交房、烂尾楼等问题。大力培育住房租赁和二手房市场，提高库存商品房利用率。规范房地产市场秩序，合理引导市场预期，促进房地产市场健康发展。

（三）坚定不移推动和谐发展，加快建设幸福城市。和谐发展是高质量发展的重要体现。要坚持以人民为中心，始终把关乎群众利益的事放在心上、抓在手上、扛在肩上，时刻和人民想在一起、站在一起、干在一起，顺民意、解民忧、惠民生，团结带领全市人民共同创造更加美好幸福的生活。

打好精准脱贫攻坚战。深入实施脱贫富民战略三年行动计划。深化闽宁协作，实施第三方项目扶贫机制，推动产业、劳务、人才等要素精准对接，合力打造“四个示范点”，加快闽宁镇从脱贫向富民转变。突出“六个精准”，坚持扶贫和扶志、扶智相结合，聚焦“两不愁、三保障”目标，紧盯月牙湖乡等集中连片贫困地区，持续推进贫困村提升、产业扶贫提质增效等重点工程，确保19个贫困村8515人脱贫出列，生态移民人均可支配收入持续增长。坚决抓好扶贫领域突出问题整改，扎实做好建档立卡动态调整和“回头看”工作。做好符合条件的自主迁徙居民脱贫工作，着力解决收入水平略高于建档立卡贫困户群众的发展问题，发挥好深度贫困人口专项救助基金作用，确保小康路上一个都不少，共富路上一个都不掉队。

千方百计扩大就业。坚持把稳就业摆在突出位置，完善就业创业政策服务体系，保障居民收入水平持续增长。全面落实创业担保贷款和各类就业创业补贴扶持政策，突出抓好高校毕业生、农村转移劳动力、退役军人、城镇困难人员等重点群体就业，城乡劳动力职业技能、创业能力培训1万人以上，转移农村劳动力8万人以上，新增城镇就业4.5万人以上，城镇登记失业率控制在4%以内。全面治理拖欠农民工工资问题。加大对就业困难人员救助力度。帮助督促停产、搬迁等企业做好职工安置工作。大力推进“双创”行动，积极培育创新创业孵化平台，发展服务业新业态、新模式，以创新带创业促就业稳增收，让群众挣钱的路子更宽些、腰包更鼓些。

提高社会保障水平。深化医疗保险制度改革，完善付费方式改革政策，优化基本医保、大病保险与健康扶贫等“一站式”结算模式。稳步提高城乡居民、城镇职工养老、医疗保障水平，继续推进被征地农民参加养老保险。推进棚户区改造，开展老旧小区供暖管网改造和加装电梯试点，让更多中低收入家庭安居宜居。大力发展养老服务事业，改革日间照料中心运营体制，完善养老护理体系。引入社会力量参与社会救助，提高城乡低保标准。实施母亲健康快车等项目，加强困难残疾人照护和生活救助，倾情倾力做好托底工作，让每一个身处困境者都得到关爱和温暖。

办好人民满意教育。深化京银教育合作，加快推进“互联网+教育”行动计划和“教育信息化2.0行动”。优化教育资源布局，推进学前教育三年行动计划，新建11所幼儿园，幼儿园普惠率达到78%。推动义务教育优质均衡发展、高中教育特色化发展，深化职业教育校企合作、产教融合，加强特殊教育，推动宁夏幼专自治区一流学科建设。清理整顿违规违法办学行为，加强民办学校规范管理，促进民办教育有序健康发展。实施一批中小学校建设项目，力促北师大银川学校幼儿园、小学建成投用，着力解决群众反映强烈的“乡村弱”“城市挤”“班额大”“负担重”等问题，力争让每个孩子都能享有公平而有质量的教育。

推进健康银川建设。加快“互联网+医疗健康”示范区建设，实施大数据共享平台、多级综合远程诊疗体系等重点项目，推进国家医疗大数据中心和产业园建设。深化与北京医疗卫生机构合作，统筹智慧医疗、医联体、急救一体化建设，加快实施市中医院迁建、妇幼保健院产科大楼等项目，建设专科诊疗中心、区域医疗中心，让优质医疗资源惠及更多群众。深化医药卫生体制改革，加强现代医院管理，推进分级诊疗、家庭医生签约服务，控制医疗费用不合理增长。优化生育全程服务，加强重大疾病防控。做好永宁县、西夏区综合医改试点工作。强化食品药品安全监管，争创国家食品安全示范城市。广泛开展体医融合及全民健身活动，举办第三届“丝绸之路”中国银川国际马拉松赛。加快国家健康城市、医养结合试点城市建设，持续开展健康村镇、健康社区等健康“细胞”创建活动，促进健康事业、健康产业互动发展，为全市人民提供全方位全周期健康服务。

做好民族宗教工作。持续加强马克思主义民族观宗教观宣传教育，全面贯彻党的民族政策和宗教工作基本方针，深入开展民族团结进步“八进”活动，加强各民族交往交流交融，不断增强“三个离不开”思想和“五个认同”意识，铸牢中华民族共同体意识，着力打造全国民族团结进步示范市。坚持宗教中国化方向，依法管理宗教场所、人员和活动，营造和谐健康的宗教关系。严厉打击和坚决抵御境外宗教渗透活动，营造和顺平静、和谐相处的社会氛围。

（四）坚定不移推动宜居发展，加快建设西北地区重要中心城市。宜居是高质量发展的重要标志。要深入贯彻区域协调发展战略，推进新型城镇化与乡村振兴互促共融，促进生产要素双向流动，着力构建优势互补、全面联动的区域发展新格局，让城乡群众在共建共享中享受美好生活。

加速银川都市圈建设。与“两市一地”增进共识、统一行动，完善都市圈基础设施、产业发展等专项规划。以交通为重点，统筹推进铁路、公路、网络、能源等基础设施互联互通，加快建设银西高铁、火车站西站房及西广场改造提升和交通枢纽地下连通工程、西线供水、智慧城市配电网等重点项目，构筑内通外联新格局。突出协同互补、错位发展，探索建立联合招商、异地开发、利税共享的协同机制，创新“研发设计在银川、转化生产在周边”的产业合作模式，打造跨区域重点产业集群。坚持与邻为伴、系统治理，推进生态环境联防联治，打造鄂尔多斯台缘、贺兰山东麓、黄河生态廊道，建设“城园一体、绿轴串联”生态绿网体系。坚持便民惠民，打破城市界线，放大“首都带首府·首府带县乡村”教育医疗合作效应，拓展延伸银川集团化办学、联合办医范围，推动公共服务共建共享。加快都市圈核心区建设，支持兴庆区打造创新发展示范区、金凤区打造对外开放高地、西夏区打造科技新城、贺兰县加快新旧动能转换、永宁县打好“翻身仗”、灵武市重构产业发展优势，推动县域经济高质量发展。

实施乡村振兴战略。深入实施乡村振兴战略三年行动计划，加快推进农业农村现代化。深化农业供给侧结构性改革，实施乡村产业富民工程，推动“1+2+4”特色产业品牌发展，支持建设有机绿色标准化生产基地，新增有机蔬菜、稻渔种养基地各1万亩，规模养殖化率达到80%以上，打造市辖区以花卉园艺、休闲观光等新业态为主的绿色生态农业发展区，两县一市以水稻、瓜菜、水产等产业为主的优质粮食生产功能区和市郊特色农产品优势区。办好第二届“中国农民丰收节”，加快苏宁现代农业产业科技园、银川枸杞田园综合体等重点项目建设，促进一二三产融合发展。稳步推进农村集体产权制度改革，支持适度规模经营，培育壮大龙头企业，鼓励新型经营主体与小农户建立契约型、股权型利益联结机制，加强职业农民和实用技术培训，激活农业农村发展活力。加快特色小城镇、美丽乡村建设，推进农村人居环境整治三年行动，突出抓好“两治两改”，加快中心村污水集中处理、农村垃圾分类和资源化利用，实施农村电网升级改造，推动道路、通讯、天然气、污废处理等基础设施城乡联网。健全自治、法治、德治相结合的乡村治理体系，培育文明乡风、良好家风、淳朴民风。

着力完善城市功能。尊重城市发展规律，推动城市发展由外延扩张向内涵提升转变，实现精明增长。精心规划城市。加快完成《银川市空间规划（2016-2035）》《银川市城市总体规划（2018-2035）》等编制，推动城市南北部等重点片区详细规划，严控楼层高度和建筑密度，优化城市色彩和天际线，加强通风廊道建设，留住观山望水的“视廊”，凸显“塞上湖城”特色。推动两县一市规划与全市规划有效衔接。强化规划执法管理，严格落实各项强制性标准和要求，维护规划权威性、严肃性和持续性。精致建设城市。加强城市特色风貌研究，推进全国城市设计试点和“城市双修”工作，大力疏解老城区功能，加强城市新片区特色建设，推进建筑风格与历史文化相融合。精心抓好银川火车站等片区城市设计和建设，改造一批特色街区。加强城市绿化美化亮化改造，建设一批小微公园，推进社区体育公园、公共场所母婴设施建设，让城市更有温度。持续开展“疏堵提畅”工程，实施亲水北大街、宏图街、沈阳路等改扩建工程，改造一批小街巷，新建一批停车场，推进城乡公交一体化，有效缓解“交通拥堵”“停车难”等问题。精细管理城市。深化“以克论净、深度保洁”模式，提升生活垃圾分类示范城市建设水平。加强智慧化管理，像绣花一样治理城市。严控新增城镇建设用地规模，实行增量供给与存量挖掘相结合的供地、用地政策，推动城市土地节约集约利用；整合优化城市资源，引导各类资本投入城市建设运营，实现城市共治共管、共建共享。

提升城市文明水平。加强公共文化服务体系建设，新建一批村级（社区）综合性文化服务中心，深化“最美银川人”“书香银川·银川书香”、志愿服务等活动，持续抓好“四送六进”“农民文化大集”等文化惠民工程，办好互联网电影节、文化创意节、黄河艺术节等活动，推动西夏陵申遗和贺兰山岩画5A景区创建，加快文化艺术精品创作和走出去步伐，为人民群众提供丰富的精神食粮。推动永宁、灵武创建全国县级文明城市，广泛开展文明村镇、文明家庭等创建活动。积极培育和践行社会主义核心价值观，加强社会公德、职业道德、家庭美德、个人品德教育，培育积极向善、创新实干、奋发有为的精神风貌。

扎实推进社会治理。加强退役军人服务管理，深入开展双拥共建、军地共建，争创全国双拥模范城市九连冠。深化“平安银川”建设，深入开展扫黑除恶专项斗争，加快“雪亮工程”建设，织密社会治安立体防控网。严格落实安全生产责任制，增强智慧安监防控能力，牢牢守住安全底线。坚持和发展新时代“枫桥经验”，加强信访法治化建设，完善社会矛盾排查预警、重大决策社会稳定风险评估、矛盾纠纷多元化解机制，引导社会组织依法参与社会治理，开展群众反映强烈突出问题“清零”行动，做到人民有所呼、政府有所应。

三、加强政府自身建设

新时代孕育新梦想，新使命呼唤新担当。作为新时代的追梦人，打铁必须自身硬，我们要切实加强政府自身建设，全面提升政府治理现代化水平，努力建设人民满意政府。

（一）坚持党的领导，建设学习型政府。坚持以党的政治建设为统领，扎实开展“不忘初心、牢记使命”主题教育，深入学习贯彻习近平新时代中国特色社会主义思想，持续开展习近平总书记视察宁夏重要讲话和重要指示批示精神“回头看”，用“学习强国”激发学习力量，进一步提高政治站位，树牢“四个意识”，坚定“四个自信”，坚决做到“两个维护”，坚决贯彻落实党中央、国务院，自治区党委、政府和市委各项决策部署。巩固提升“三转一高”大学习大讨论成果，深化新发展理念、现代化经济体系、供给侧结构性改革等内容学习，学以致用、学用结合，增强专业能力，倡导专业精神，努力增强“八种本领”，切实提升推动高质量发展的履职水平。

（二）坚持依法行政，建设法治型政府。推进法治政府建设，依法行使职权、履行职责、开展工作，扎实开展“七五”普法，深入推进科学立法、严格执法、公正司法、全民守法。修订环境保护、城乡建设、历史文化保护等领域政府规章，强化规范性文件监督管理。坚持重大决策公开听证、专家论证、合法性审查、社会风险评估和集体研究决定，增强政府治理精准度和有效性。自觉接受人大法律监督、工作监督和政协民主监督，主动接受社会公众监督和舆论监督，高质量办理人大议案建议和政协提案，定期向人大、政协通报重要工作。加强诚信政府建设，强化守信激励失信惩戒机制应用。全面推进政务公开，做好政策解读回应，推动“政府开放日”制度化常态化。

（三）坚持勤政为民，建设实干型政府。坚持实干兴银，鼓精气神、做精细活。大兴调查研究之风，问政于民、问需于民、问计于民；大兴干事创业之风，敢于担当、敢于较真、敢于碰硬，责任面前不推诿，问题面前不回避，困难面前不退缩，把工作当事业干，定了就干、干就干好，务求各项工作落到实处、取得实效。健全以高质量发展为导向的效能考核评价体系，以实绩论英雄。改进和规范督查检查考核工作，为干部减压、为基层减负。打造“市长在线”政民互动平台，进一步发挥“电视问政”“12345”监督作用，坚决整治“嘴上空跑”“纸上旅行”“虚多实少”等问题，对懒政庸政者追责问责。进一步关心关爱干部，落实正向激励和容错纠错机制，旗帜鲜明地为实干担当者撑腰鼓劲，激励公职人员尽职尽责、苦干实干，以干部的“辛苦指数”赢得群众的“幸福指数”。

（四）坚持从严治政，建设廉洁型政府。牢固树立“100-1=0”的廉洁从政理念，认真履行党风廉政建设主体责任和“一岗双责”，严格落实中央八项规定及其实施细则精神和自治区、银川市若干意见，坚决纠正“四风”新表现，巩固中央巡视反馈问题整改落实成果。牢固树立过“紧日子”的思想，强化预算刚性约束，严控超规模、超标准现象，一般性项目支出和公用经费分别压缩50%和20%，把有限的财力用在民生改善和重点项目建设上。强化审计监督，严格监管工程建设、招标投标、征地拆迁、产权交易、政府采购等重点领域、重点环节，严格领导干部经济责任审计和“收支两条线”管理，从源头上防止腐败行为发生，努力营造政府系统风清气正的政治生态。

各位代表！使命呼唤担当，奋斗创造幸福。让我们更加紧密地团结在以习近平同志为核心的党中央周围，在自治区党委、政府和市委的坚强领导下，振奋精神、主动作为、攻坚克难，以坚如磐石的信心、只争朝夕的劲头、坚韧不拔的毅力，一步一个脚印，推动银川高质量发展，为实现“两个率先”目标、“建设美丽新宁夏、共圆伟大中国梦”而努力奋斗，以优异成绩迎接中华人民共和国成立70周年！
